# Recognizing thrombotic thrombocytopenic purpura beyond the pentad

**DOI:** 10.1093/omcr/omag168

**Published:** 2026-09-14

**Authors:** Olivia M Pupiec, Julie R Levin, Christopher N Cooley, Diane Paratore

**Affiliations:** Department of Emergency Medicine, Corewell Health Farmington Hills, 28050 Grand River Avenue, Farmington Hills, MI 48336, United States; Michigan State University College of Osteopathic Medicine, 965 Wilson Road, East Lansing, MI 48824, United States; Department of Emergency Medicine, Corewell Health Farmington Hills, 28050 Grand River Avenue, Farmington Hills, MI 48336, United States; Department of Emergency Medicine, Corewell Health Farmington Hills, 28050 Grand River Avenue, Farmington Hills, MI 48336, United States; Michigan State University College of Osteopathic Medicine, 965 Wilson Road, East Lansing, MI 48824, United States; Department of Emergency Medicine, Corewell Health Farmington Hills, 28050 Grand River Avenue, Farmington Hills, MI 48336, United States; Michigan State University College of Osteopathic Medicine, 965 Wilson Road, East Lansing, MI 48824, United States

**Keywords:** thrombotic thrombocytopenic purpura, joint pain, ADAMTS13, Schistocytes

## Abstract

Thrombotic thrombocytopenic purpura (TTP) is a rare, life-threatening hematologic disorder characterized by widespread microvascular thrombosis. Classic clinical features include: fever, microangiopathic hemolytic anemia, thrombocytopenia, renal dysfunction, and neurologic abnormalities, such as altered mental status, confusion, or coma. Prompt recognition and initiation of plasma exchange therapy in the emergency department (ED) are essential for patient survival. We report a case of a 40-year-old female who presented to the ED with a vague complaint of joint pain and no remarkable findings on physical examination. Despite the absence of hallmark features, further evaluation revealed a diagnosis of TTP. This case underscores the diagnostic challenge posed by atypical TTP presentations and highlights the importance of maintaining a high index of suspicion, even in the absence of classic clinical signs.

## Introduction

Thrombotic Thrombocytopenic Purpura (TTP) is a rare, life-threatening blood disorder that causes widespread clotting in small blood vessels due to severe deficiency of the enzyme ADAMTS13 [1, 2]. This enzyme regulates clotting by breaking down the von Willebrand Factor, a protein involved in platelet adhesion [2]. Without sufficient ADAMTS13 levels, excessive clots form, leading to low platelet counts (thrombocytopenia), bruising (purpura), and organ damage [2]. Symptoms often appear suddenly and include fatigue, fever, confusion, shortness of breath, anemia, and kidney issues. TTP presents in approximately 11 cases/million people [3]. It can be inherited or acquired, and is more common in women, African Americans, and people aged 20–50 [2]. Triggers included autoimmune diseases, pregnancy, certain medications, cancer, and infections [1]. Prompt diagnosis using blood tests and plasmapheresis is critical to prevent severe complications such as cerebral vascular accident, coma, or myocardial infarction [2].

**Table 1:** Labs on arrival.

**Test**	**Patient Value**	**Reference Range**
WBC	9.0/L	3.3–10.7 ×10*9/L
RBC	3.65	3.87–5.08 ×10*12/L
Hemoglobin	9.0 g/dL	12.1–15.0 g/dL
Hematocrit	27.5%	35.4–44.2%
MCV	75.3 fL	79.5–100.4 fL
RDW	22.2%	11.5–15.4%
**Platelets**	**17 × 10** ^**9**^ **/L**	150–400 × 10^9^/L

## Case report

A 40-year-old African American woman with a medical history of fibromyalgia, iron deficiency anemia, sarcoidosis, hypertension, PTSD, and asthma presented to our community emergency department (ED) with one month of worsening joint pain in her wrists and ankles. She denied any trauma or change in activity. Acetaminophen and ibuprofen did not provide any relief. She also reported chronic fatigue, intermittent numbness, tingling in her hands and feet, and a new brown, oval discolorations on her fingers and toes.

One week prior, a rheumatologic workup, including ESR, CRP, SSA/SSB, and rheumatoid factor, was negative. She reported that her fibromyalgia had previously been controlled, and that her current pain was distinct from that at baseline. Otherwise, review of systems was negative. Her daily medications included albuterol, vitamin D, bupropion, folic acid, ferrous sulfate, gabapentin, clonazepam, cyclosporine, meloxicam, methylphenidate, pantoprazole, and montelukast.

Initial vital signs on arrival were as follows: temperature, 36.4°C; pulse 82; respiratory rate 18, blood pressure, 159/63 mmHg; SpO₂ 100%.

### Physical exam

General: patient was alert, tearful, and non-toxic.

Skin: warm with normal capillary refill. No active bruising, petechiae, or purpura were noted. Oval brown macules were noted on the palmar surface of the left thumb and plantar surfaces of the feet, which were non-tender, and without erythema. The remaining examination was unremarkable.

**Urinalysis:** 3+ blood, 300 mg/dL protein, and > 20 RBC/HPF.

**Peripheral smear:** polychromasia, 1–2 schistocytes/HPF, bite cells, and decreased platelets.

**Lactate dehydrogenase:** 803

**Haptoglobin:** < 8

**Bilirubin:** 3.0

Basic metabolic panel, HCG, and syphilis tests were normal. The patient was administered intravenous fluids, acetaminophen, and morphine for pain. Thrombocytopenia with schistocytes on smear prompted testing for LDH, haptoglobin, and ADAMTS13. Hematology was consulted, and high-dose intravenous methylprednisolone (1000 mg daily) was initiated for possible ITP versus TTP. A right internal jugular hemodialysis catheter was placed in anticipation of the plasmapheresis. Chart review revealed chronic anemia (Hgb 9–10 g/dL since 2021).

ADAMTS13 activity later returned < 5%, confirming TTP. She underwent daily plasmapheresis until the platelet counts exceeded 100 × 10^9^/L, after which treatments were spaced to every other day. She was hospitalized for 22 days and discharged with full resolution of joint pain and normalization of laboratory findings.

## Discussion

Thrombotic thrombocytopenic purpura (TTP) is a rare, life-threatening hematologic emergency characterized by widespread microvascular thrombosis due to a severe deficiency of ADAMTS13, a von Willebrand factor (vWF) cleaving protease [3]. While its classic pentad includes thrombocytopenia, microangiopathic hemolytic anemia, renal dysfunction, neurologic abnormalities, and fever, many patients do not present with all five features, making diagnosis challenging [4].

TTP has a reported mortality rate of 20% if it is not promptly identified and treated [3]. Certain populations are at a higher risk, including: women, African Americans, and those residing in urban settings [3]. A patient’s presentation that does not follow the classic pentad often leads to misdiagnosis, especially when symptoms mimic more common conditions such as immune thrombocytopenic purpura (ITP); disseminated intravascular coagulation (DIC); systemic autoimmune diseases; hemolysis, elevated liver enzymes, and low platelets (HELLP) syndrome; malignant hypertension; infections; antiphospholipid syndrome; and adverse drug reactions [5].

The evaluation of TTP should include: a complete blood count (CBC), peripheral blood smear, bilirubin, LDH, Coombs test, kidney function test, urinalysis, and ADAMTS13 assay [1]. Early identification is critical for these patients, but the decision to initiate plasmapheresis can be difficult given the rarity of TTP [6]. Plasmapheresis is s treatment for TTP and is aimed at removing circulating antibodies while replenishing ADAMTS13 levels [1]. Typically, plasmapheresis is continued daily until organ dysfunction resolves, platelet counts stabilize, and signs of hemolysis subside [1]. Additional therapies include caplacizumab, which inhibits platelet aggregation and microvascular clot formation, and immunosuppressive agents such as prednisone, rituximab, cyclophosphamide, and cyclosporine A [1]. In rare, refractory cases, splenectomy may be considered as well [1].

Notably, patients who survive an acute episode of TTP remain at risk of relapse and long-term complications [5]. It is crucial for these patients to continuously monitor ADAMTS13 activity and anti-ADAMTS13 antibody titers as persistent immune complexes and low enzyme activity has been shown to predict the recurrence of this disease [5]. This emphasizes the need for long-term follow-up and the contemplation of preventative therapy in high risk individuals [5].

Our patient presented with joint pain, an atypical initial symptom of TTP. As physicians, we are taught to recognize the classic pentad of TTP; however, this case demonstrates that a full pentad is not required to establish the diagnosis. Her physical examination was unremarkable, which could have led to a missed diagnosis of her condition. However, laboratory studies had revealed key diagnostic features: severe thrombocytopenia, anemia with schistocytes on peripheral smear, decreased haptoglobin, elevated LDH, and increased bilirubin levels. A confirmatory ADAMTS13 assay showed activity of < 5%, supporting the diagnosis of TTP. This case highlights the necessity of maintaining a broad differential diagnosis in patients with nonspecific or vague complaints. To our knowledge, this is the first case report to document joint pain as the primary presenting symptom of TTP.

## Consent

Informed patient consent was obtained.

## Guarantor

Julie R. Levin, DO.
